# A randomised controlled trial and mediation analysis of the ‘Healthy Habits’, telephone-based dietary intervention for preschool children

**DOI:** 10.1186/1479-5868-10-43

**Published:** 2013-04-08

**Authors:** Amanda Fletcher, Luke Wolfenden, Rebecca Wyse, Jenny Bowman, Patrick McElduff, Sarah Duncan

**Affiliations:** 1 School of Psychology, University of Newcastle, Newcastle, NSW, Australia; 2 School of Medicine and Public Health, University of Newcastle, Newcastle, NSW, Australia; 3 Priority Research Centre Health Behaviour, University of Newcastle, Newcastle, NSW, Australia; 4 Priority Research Centre Physical Activity and Nutrition, University of Newcastle, Newcastle, NSW, Australia; 5 Hunter Medical Research Institute (HMRI), Newcastle, NSW, Australia; 6 Hunter New England Population Health, Locked Bag 10, Wallsend, NSW, 2298, Australia

## Abstract

**Background:**

Consumption of non-core foods in childhood is associated with excessive weight gain in childhood. Parents play a vital role in establishing healthy diet behaviours in young children. The aim of this study was to assess the effectiveness of a telephone-based intervention in reducing child consumption of non-core foods, and to examine parent and home food environment mediators of change in child consumption.

**Methods:**

The ‘Healthy Habits’ trial utilised a clustered randomised controlled design.

**Setting/participants:**

Parents were recruited from 30 preschools (N = 394 participants, mean age 35.2±5.6 years). Parents randomized to the intervention group received four telephone contacts and print materials. Parents allocated to the control condition receive generic print materials only. Non-core food consumption was assessed using a validated child dietary questionnaire at baseline, 2 and 6 months post recruitment in 2010.

**Results:**

The intervention was effective in reducing child consumption of non-core foods at 2 months (intention to treat analysis: z=-2.83, p<.01), however this effect was not maintained at 6 months. Structural equation modelling using 2 month data indicated that child access to non-core foods in the home and child feeding strategies mediated the effect of the intervention.

**Conclusion:**

The telephone-based intervention shows promise in improving short term dietary behaviour in preschool age children, however further development is needed to sustain the effect in the long-term.

**Trial registration:**

Australian Clinical Trials Registry: ACTRN12609000820202

## Background

Childhood obesity has significant health consequences, including increased risk of developing chronic diseases such as type 2 diabetes and cardiovascular complications [1]. The psychological and behavioural impacts are also well documented, and include bullying, social isolation, poor self-esteem, and disordered eating [2,3]. Given the likelihood of excessive weight persisting from childhood into adulthood [4], interventions targeting the prevention of excessive weight gain in childhood have been recommended to avert the future health burden of obesity [5].

An important determinant of child weight status is the consumption of ‘non-core foods’, that is, foods that are high in fat, salt or sugar [6]. The positive association between excessive consumption of non-core foods and obesity in children and adults is well established [7]. Similarly, interventions targeting reductions in the intake of non-core foods have been found to be effective in preventing excessive weight gain in children [8]. Accordingly, recommendations for the prevention of overweight and obesity encourage diets consistent with healthy eating guidelines which state that children only consume non-core foods occasionally, and in small amounts [9].

Early childhood represents an ideal opportunity for dietary intervention as preschool-age children are imitative of the dietary behaviours and eating patterns of their parents and authority figures, and as dietary attitudes and behaviours established during this period often persist into adulthood [4]. Furthermore, there appears to be capacity to influence young children’s food preferences through the social-affective context in which foods are offered [10]. A number of parent and home food environment factors are thought to be particularly influential to the development of a child’s diet [11]. The availability and accessibility of non-core foods are necessary prerequisites for their consumption and are unsurprisingly positively associated with child non-core food consumption [12]. However, restricting access can increase child preference for and intake of non-core food in the absence of parental monitoring [13]. Parental pressure for children to eat, and the use of strategies that focus children’s attention on rewards or punishments, consuming all on their plate, or television viewing, is thought to hinder the development of child self regulation of their eating, particularly in response to energy dense foods [12-15]. The use of non-core food rewards can also adversely impact on child diet through reinforcing child preference and liking for the food reward [16]. However, role modelling healthy food habits by eating meals together as a family at the dinner table [14], and high parental self-efficacy regarding child feeding has also been suggested to be positively associated with healthier child diet [17,18].

Given the relationships between parent and home environment characteristics, interventions targeting such characteristics have been recommended to improve child diet and reduce the risk of excessive weight gain [4,19]. Currently, however, few trials have been conducted examining the effectiveness of interventions incorporating parent and home environment strategies. Three recent systematic reviews found a total of only eight nutrition-based obesity prevention studies targeting parents of preschool-aged children [16,18,20]. The studies varied considerably in terms of intervention setting, intensity, delivery modalities and the parent and home food environment characteristics targeted, limiting the capacity for researchers and practitioners to identify the components of effective initiatives.

Mediation analysis allows researchers to identify mechanisms by which one variable influences another [21], can assist in understanding the causal pathways by which interventions operate [22], and has been recommended as a means of improving the design and effectiveness of future obesity prevention interventions [23]. As such, investigating the mediators of effective interventions that target parent and home food environment characteristics may allow an assessment of how such interventions are effective, and how they may be improved. Despite recommendations that mediation analysis be routinely used in intervention studies [24], its application in childhood obesity research is in its infancy. To our knowledge there have been no published mediation studies of interventions targeting dietary outcomes of pre-school aged children. A recent systematic review of interventions that examined mediators of dietary behaviour change in 5-18 year olds identified just seven studies [25]. The review found no convincing evidence for any of the investigated mediating variables, but highlighted the limitations of the included studies such as low power, insensitive measures, and ineffective intervention strategies.

‘Healthy Habits’ is a randomised controlled trial of a telephone-based parent intervention targeting parent and home food environment characteristics as a means of improving child diet [26]. The aim of the present study was to assess the effectiveness of the intervention in reducing child consumption of non-core foods, a secondary outcome investigated in the trial. We also sought to conduct an exploratory analysis of the causal mechanisms of this dietary change through mediation analysis.

## Methods

### Study design

**Figure 1:**
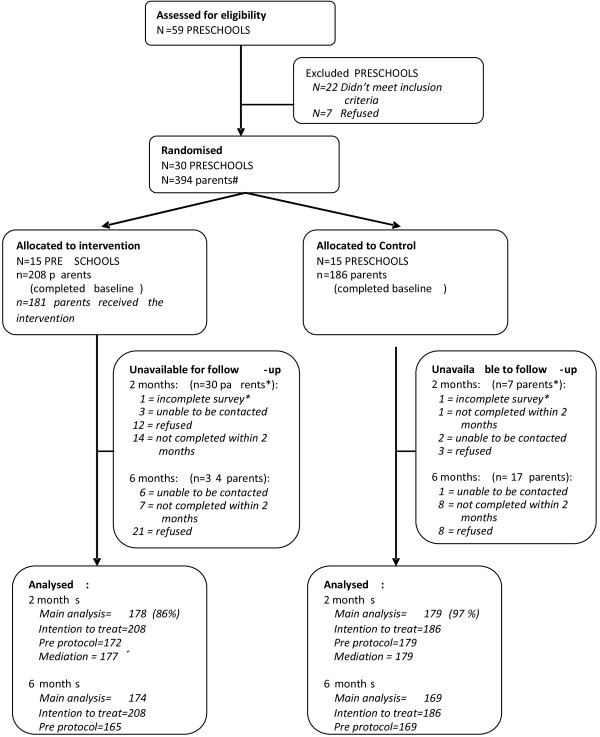
**Consort diagram describing participant flow through the trial. **# Approximately 2200 parents were invited to participate, 418 consented and 178 refused to participate. Of the 418 consenting parents, 24 were not randomised because they were subsequently unable to be contacted (n=5), didn’t meet inclusion criteria (n=9), or refused to complete the baseline survey when contacted (n=10). 2 parents were excluded from the 2month analysis as they had been away from their child for the past 24 hours and/or past 7 days, and were unable to answer questions about their child’s non-core food consumption. +1 participant only partially completed the baseline survey. They completed the NCFI data, but mediator data was not collected, hence they were included in the main analysis, but not the mediation analysis.

The ‘Healthy Habits’ study utilised a cluster randomised controlled design (Figure 1). The trial was registered with the Australian Clinical Trials Registry (ACTRN12609000820202). This paper reports 2 and 6 month trial outcomes collected via telephone interview with parents.

### Participants

Parents were recruited through preschools within four Local Government Areas of the Hunter region of New South Wales, Australia. Preschools were excluded from participation if they provided food to children, exclusively catered for children with special needs, or had been involved in child healthy eating research projects within the preceding months. At eligible and consenting preschools, information and consent forms were distributed via methods considered most appropriate by the preschool supervisor, which usually involved research staff attending preschools to distribute recruitment packs to parents and to be available to answer parent questions. Parents were eligible to participate if they: had a 3-5 year-old child who resided with them for 4 or more days per week; were responsible for providing food to their child at least half of the time; had a child with no dietary requirements that would make Australian fruit and vegetable intake recommendations unsuitable; and if they were literate in English.

### Randomisation and allocation

Preschools were randomly assigned to intervention (n=15) and control (n=15) conditions using a randomisation function in Microsoft Excel. Preschools were stratified by high and low socio-economic status based on the area in which they were located [27] and were randomised in a 1:1 ratio (intervention:control) in randomly sequenced blocks of between two and six preschools. The randomisation was conducted by a statistician not otherwise associated with the study.

### Intervention

To maximise potential public health utility, the trial sought to test a relatively brief intervention. Parents allocated to the intervention condition received four, 30 minute telephone calls, from an experienced health interviewer who delivered a pre-written script using a Computer Assisted Telephone Interview (CATI) system. Telephone-based interventions of similar intensity have been found to be effective in changing health behaviours among adults [28,29]. One call was scheduled each week for one month. While the intervention primarily targeted increasing child consumption of fruit and vegetables, a key focus of the intervention was reducing the consumption of non-core foods which can potentially displace fruit and vegetables in a child’s diet. The intervention was developed by an advisory group consisting of psychologists and dieticians and utilised a range of behavioural change techniques [30]. In addition to the phone calls, participants received a comprehensive workbook with relevant factsheets and activity tracking sheets, a pad of meal planners, and a cookbook as part of the intervention package.

The content of the telephone support drew on socio ecological theory and the family-based theoretical framework proposed by Golan and Weizman [19] for childhood obesity treatment. The framework integrates behavioural, social learning and family systems theory and focuses on changing parental cognitions and behaviour, as well as the home environment. Specifically, the script addressed three key areas:

i) Parent role-modelling - consuming healthy foods (such as eating fruits and vegetables in place of non-core foods) in view of children during meals and snacks;

ii) The availability and accessibility of foods in the home - making healthy foods readily accessible in the home, and preparing and presenting them in ways which may be appealing for children. Parents were encouraged not to bring non-core foods into the home, or to store these out of sight of children;

iii) Supportive food routines – parents were encouraged to introduce routines such as eating dinner as a family, at a table, and without the television on.

The intervention calls incorporated a number of behaviour change techniques (as classified by the taxonomy of Abraham and Michie) to support participants to improve such parent and home food environment characteristics [30]. Behaviour change techniques included: providing information on consequences, encouragement, instruction and feedback; prompting barrier identification, review of goals and self-monitoring; and teaching to set goals, undertaking graded tasks and time management. Particular attention was paid to the improvement of parent self-efficacy in implementing positive child feeding practices and modifying the home environment through; goal setting, assisting parents to overcome barriers to change, prompting review of behavioural goals, providing general encouragement, and reinforcing goal attainment [31]. Similarly, specific strategies were implemented to encourage parents not to employ pressure or controlling feeding practices. These strategies included the use of the division of responsibility framework which teaches parents that their role is to plan, prepare and provide a variety of nutritious foods to their child, but that their child then has autonomy in choosing if, what and when to eat the foods provided [32]. Intervention delivery occurred from April to December, 2010.

### Control group

Parents allocated to the control group were mailed a generic print booklet (The Australian Guide to Healthy Eating). The booklet contains basic nutrition information and recommendations for a healthy diet for adults and children. It includes a section on ‘extra foods’ (foods high in fat, salt or sugar) in which such foods are recommended to be consumed infrequently or in small amounts. The booklet also contains a small number of healthy recipe ideas and suggestions [32]. (The resource can be downloaded at (http://www.health.gov.au/internet/main/publishing.nsf/Content/E384CFA588B74377CA256F190004059B/$File/fd-cons.pdf). Control group participants had no further contact until follow-up data collection.

### Data collection

All data collection occurred via telephone by trained interviewers who were blind to participant group allocation. The survey contained standard scripted items and was administered using a computer assisted telephone interview. Participating parents were called approximately one week following recruitment to complete the baseline survey and then approximately 2 and 6 months later for follow-up data collection. The baseline survey was conducted from April to October 2010.

### Measures

#### Children’s Non-core food consumption

Child consumption of non-core foods was assessed using the non-core foods (NCF) subscale from the Children’s Dietary Questionnaire (CDQ) [6]. This subscale is based on the frequency of child consumption of non-core foods within the past seven days, from commonly consumed categories of non-core foods. It provides a continuous outcome scored from 0 to 10.3, with a higher score indicating greater consumption of non-core foods, and a score above 2 indicating that the child’s diet exceeds recommended amounts of non-core foods based on the Australian dietary guidelines. The NCF subscale has established test-retest reliability (intraclass correlation coefficient = 0.90), validity (spearman correlation = 0.31) and internal consistency (alpha = 0.56) on a comparable sample of Australian children, and has been recommended for use in intervention research [6].

#### Hypothesised mediating variables

**Non-core food availability and accessibility within the home **The extent to which non-core foods were available and accessible to children in their homes (‘access’) was assessed using items from the Healthy Home Survey (HHS) [33]. The HHS assesses features of the home food environment thought to influences weight-related behaviours of children. Each item on the HHS has been individually assessed for reliability (test re-test) by comparing telephone survey responses of parents (n=85) of children 3-8 years at one week intervals using the Kappa statistic. Validity of HHS items was also assessed by comparing parental response to HHS items with a home observation performed within 14 days of the telephone survey [34] using the Kappa statistic. The Kappa statistic provides a standardised quantitative measure of the magnitude of agreement between two assessments above chance [35]. A Kappa of 0 represents agreement expected due to chance. To assist interpretation of Kappa statistic, Landis and Koch (1977) suggest a Kappa of >0-0.2 represents ‘slight agreement’; 0.21-0.40 ‘fair agreement’; 0.41-0.60 ‘moderate agreement’; 0.61-0.80 ‘substantial agreement’; 0.81-0.99 ‘almost perfect agreement’; and 1 ‘perfect agreement’ [36]. The current study utilised four items assessing availability of non-core foods in the home: salty snacks (test re-test reliability prevalence and bias adjusted kappa (PABAK) = 0.91; validity PABAK = 0.93); sweet snacks (reliability PABAK = 0.91, validity PABAK = 0.88); confectionery (reliability PABAK = 0.77, validity PABAK = 0.73) and soft drinks (reliability Kappa = 0.63, validity Kappa = 0.54); and four items assessing the child’s access to these non-core foods: salty snacks (reliability Kappa=0.68, validity Kappa=0.07); sweet snacks (reliability Kappa=0.62, validity Kappa=0.29); confectionery (reliability Kappa=0.72, validity Kappa=0.22) and soft drinks (reliability Kappa=0.71, validity Kappa=0.26).

**Parental self-efficacy for providing healthy foods to their child**The ‘Parental self-efficacy for child diet’ (‘PSEC’) scale was used to assess parent self-efficacy related to child feeding. This scale was developed by members of the research team in response to a lack of such measures in the literature at the time of trial commencement, and was developed based on Bandura’s guide for constructing self-efficacy scales [37]. The scale specifically measures self-efficacy of parents to provide a healthy diet for their child. The scale uses a 6-point Likert scale and consists of 10 items, 7 of which are reverse scored. Items included: ‘Providing a healthy diet for children is difficult to manage’, ‘I can solve most problems with my child’s eating habits if I invest the necessary effort’ and ‘It’s too hard to provide my child with healthy food when I’m feeling tired’. Factor analysis of scale items revealed that all items loaded into a single factor and were internally consistent (Cronbach’s alpha level = 0.735).

**Family mealtime practices **Frequency of family meals (‘family meals’) was measured by a HHS item asking how many nights each week the family usually sat together for their evening meal (reliability: kappa =0.79) [34].

**Television practices during mealtimes **Television viewing during the evening mealtime (‘TV’) was assessed by an item from the HHS which measured the number of days per week the child usually eats dinner in front of the television (reliability: kappa = 0.80) [34].

**Child feeding strategies **Items from the HHS assessed parents’ use of specific child feeding practices to influence child eating behaviours (‘strategies’). On a 5-point Likert scale (‘all of the time’ to ‘never’) parents were asked how frequently they did the following: ask their child to eat everything on their plate at dinner (reliability kappa=0.75); restrict dessert if their child does not eat the food on their plate at dinner (reliability kappa=0.61); reward their child with desserts, snacks or confectionary if they finish their dinner (reliability kappa=0.58); allow their child to eat only at set meal times (reliability kappa=0.40); and allow their child to help him/herself to snacks when at home (reliability kappa=0.65) [34].

**Pressure to eat **The ‘Pressure to Eat’ subscale from the Child Feeding Questionnaire (CFQ), demonstrated to be internally consistent (alpha = 0.70), was included to measure the extent to which parents are concerned about the amount of food eaten by their child and pressure their child to increase their intake. Specifically the four item scale assesses on a 5-point Likert scale if they believe their child should always eat all of the food on their plate; if they have to be especially careful to ensure their child has enough to eat; if they try to encourage their child to eat when they are not hungry; and if they believe if they didn’t regulate their child’s eating their child would not eat enough. A higher score indicates a greater concern and more pressure to eat [38].

### Analysis

SAS software version 9.2 (SAS Institute Inc., Cary, NC, USA) was used to perform analysis to describe the study sample and assess intervention effectiveness. Statistical tests were two tailed with an alpha value of 0.05.

#### Outcome analyses

Analysis of Covariance was used to evaluate the effectiveness of the intervention in reducing child consumption of non-core foods, that is, a linear regression model was fit to the data with NCF post treatment as the outcome, treatment group as the main predictor of interest and the analysis was adjusted for baseline values of NCF. The model was fit using a Generalised estimating equation (GEE) approach to adjust for the clustering of children within preschools. Initially the analysis was conducted including those participants with complete data at baseline, 2 and 6 months. An additional intention to treat analysis was conducted whereby participants’ baseline NCF scores were carried forward to substitute for any missing data at the 2 or 6 month follow up.

The eight items from the HHS measuring the availability and accessibility of non-core foods described above were combined to form the single variable ‘access’. For each type of non-core food (salty snacks, sweet snacks, confectionery and soft drinks) participants could score between 0 and 3 (0=not available in the home; 3=available and accessible to the child) providing a variable with scores possibly ranging from 0 to 12. Responses to the item from the HHS assessing family meals were dichotomised (1=eat a meal together every day or 2=do not eat a meal together every day) to create the single categorical variable ‘family meals’ to measure the presence (or absence) of daily family meals. Similarly the responses to the item from the HHS asking parents about eating dinner in front of the TV were dichotomised (1=no TV during dinner; 2=TV during dinner on one or more evenings). Non-normal continuous variables (e.g. days per week the family eats dinner together at a table, days per week the child eats dinner in front of the television) were dichotomised using cut points with which the higher levels of children's fruit and vegetable consumption have previously been associated [14,39,40].

#### Mediation analyses

Mediation analyses were conducted where a significant between-group intervention effect was found for the consumption of non-core foods. Analysis Of Moment Structures (AMOS 19.0; SmallWaters, Corp., Chicago, IL) Structural Equation Modelling (SEM) software was used to investigate whether the proposed mediating variables mediated the effect of the intervention on child consumption of non-core foods.

The SEM was fit using maximum likelihood approach. Model fit was assessed using multiple indices, including chi-squares index, goodness of-fit index (GFI), adjusted goodness of-fit (AGFI), normed fit index (NFI), and root mean square of approximation (RMSEA). The chi-square index tests the null hypothesis that the model is a good fit of the data, and hence if the chi-square is not significant, the model is regarded as acceptable in that the observed relationships are similar to the predicted relationships in the model. A non-significant normed chi-square (CMIN) or a CMIN value of less than 2, were considered indicative of a good fitting model [41]. The GFI and AGFI statistics provide estimates of the proportion of variance in the variance-covariance matrix accounted for by the proposed model. GFI and AGFI scores greater than 0.95 were considered acceptable [42]. A Normed Fit Index (NFI) exceeding 0.90 [43,44], and RMSEA less than 0.8 were considered acceptable [41].

Two models are presented. A ‘full’ model that provides for the most comprehensive exploration of the data, and a ‘final’ model that is the most parsimonious. To start the process of building these models, a ‘base’ model showing the relationship between group allocation and the outcome measure (NCF) was first established, and then each mediator was added to the model individually in five separate ‘simple’ models. Baseline measures for the outcome variable each and mediating variable were included in all models to adjust for differences between subjects, and co-variance between these variables was included to minimise unexplained variance in the model and to allow the pathways to be free to vary. The mediators were added to the model one at a time based on the effect size and significance of the mediating pathways and the mediation effect size in the simple models. The variables that showed the strongest significant mediation effect were added first, and the fit of the model was tested with the addition of each new mediating variable. The variables that failed to show a mediation effect in the simple models were not added to the more comprehensive model. The ‘full’ model was investigated first, and then goodness of fit measures were examined to determine the ‘final’, most parsimonious model. The magnitude of effect sizes are reported, as are the standardised path coefficients and the total percentage of variance explained by the model.

## Results

### Participants

Fifty nine preschools were approached, of which 37 were assessed as eligible and invited to participate. Thirty of the 37 eligible preschools consented to participate in the study and seven refused, yielding a service level response rate of 81%. A total of 605 parents returned consent forms; 178 declined participation, 418 provided consent (of which 9 were ineligible) and 394 participated (Figure 1). A total of 357 participants provided 2-month follow-up data, and 343 provided 6-month data. The demographic characteristics of the 394 participants who completed the baseline survey are presented in Table 1. There were no significant differences in demographic characteristics of intervention and control group participants. Control group participants who completed the 6 month follow-up were more likely to be tertiary educated (52% vs. 24%, p=0.03) and consumed fewer daily serves of vegetables (3.8 vs 3.0, p=0.01) than those who didn’t provide follow-up data at this time point. There were no other significant differences between those completing 2 or 6 month follow-up assessments within either the intervention or control group.

**Table 1:** **Characteristics of participants at baseline**[26]

	**Intervention**	**Control**
	**N=208**	**N=186**
	**%/Mean±sd**	**%/Mean±sd**
**Parent demographics**		
Age (years)	35.2±5.6	35.7±5.0
Gender – female	95.2%	96.8%
Household income ≥ $100,000	42.4%	40.2%
University education	45.2%	49.5%
Aboriginal and/or Torres Strait Islander	1.0%	3.2%
Number of children (<16 years) in household	2.3±0.8	2.3±0.7
**Child demographics**		
Age (years)	4.3±0.6	4.3±0.6
Gender – female	51.0%	45.7%
Aboriginal and/or Torres Strait Islander	1.0%	4.8%

#### Child non-core food consumption

Main analysis revealed that mean NCF scores were significantly lower in the intervention compared to the control group at 2-month follow-up (z=-2.89, p<0.01). The effect remained significant when NCF scores at baseline were carried forward for missing data at the 2-month follow-up as part of an intention to treat analysis (z=-2.83, p<0.01). These effects were not maintained at 6 months (Table 2).

**Table 2:** Intervention and control Non-core food (NCF) subscale scores (mean±standard error) at baseline and 2 and 6 month follow-up by group

**Analysis**		**Control**	**Intervention**	p*
		**Mean±SEM**	**Mean±SEM**	
Main analysis	Baseline (n=394)	2.59±0.08	2.48±0.08	
	2 month (n=357)	2.57±0.11	2.24±0.07	<0.01*
	6 month (n=343)	2.47±0.10	2.29±0.09	0.20
Intention to treat analysis	Baseline (n=394)	2.59±0.08	2.48±0.08	
	2 month (n=394)	2.60±0.10	2.27±0.06	<0.01*
	6 month (n=394)	2.52±0.09	2.34±0.06	0.22

*p<.05.

#### Model testing

Given that the intervention effect was only statistically significant at the 2-month follow-up, mediation analyses were conducted on data at this time point only. The ‘base’ model (group allocation and NCF scores, with no mediator variables) was a good fit for the data, *χ*^2^ (1, *N=356*) = 0.60, *p =* 0.44; CMIN = 0.60, GFI = 0.99, AGFI = 0.99, NFI = 0.99, RMSEA = 0.00. When mediators were added individually in separate ‘simple’ models, mediation effects were found for all mediators other than ‘family meals’ and ‘TV’ (Table 3) which were excluded from subsequent analysis. ‘Strategies’ proved to be the strongest simple model, *χ*^2^ (4, *N=356*) = 6.97, *p =* 0.14; CMIN = 1.74, GFI = 0.99, AGFI = 0.97, NFI = 0.98, RMSEA = 0.05, followed by ‘access’ *χ*^2^ (4, *N=356)* = 6.59, *p =* 0.16; CMIN = 1.64, GFI = 0.99, AGFI = 0.97, NFI = 0.98, RMSEA = 0.04. These variables were both put into the first more comprehensive model as part of the process of building the model, and the model remained a good fit for the data *χ*^2^ (10, *N=356*) = 15.37, *p =* 0.11; CMIN = 1.54, GFI = 0.99, AGFI = 0.97, NFI = 0.97, RMSEA = 0.04. The variable ‘pressure’ was the next to be added into the model, however the fit of the model worsened according to some measures of model fit, *χ*^2^ (19, *N=356*) = 48.95, *p =* 0.0; CMIN = 2.58, GFI = 0.97, AGFI = 0.93, NFI = 0.95, RMSEA = 0.07. The variable ‘PSEC’ was then added to the model, and the addition of this variable slightly improved the fit of the model to the data, *χ*^2^ (31, *N=356*) = 61.97, *p =* 0.001; CMIN = 1.99, GFI = 0.97, AGFI = 0.94, NFI = 0.94, RMSEA = 0.05. Table 3 shows the standardised regression weights of the paths in simple models, and shows that the magnitude of the direct effect of the intervention decreases from -0.27 to -0.12 as more mediators are added to the model.

**Table 3:** Regression weights of paths in the mediation models

**Model (Model number)**	**Path A**	**Path B**	**Path C**
	**Treatment allocation ➔ mediator**	**Mediator ➔ outcome**	**Treatment allocation ➔ outcome**
Base (1)			-.27**
**Simple models**			
Strategies (2)	-.39***	.14**	-.20*
Access (3)	-.36*	.13***	-.20*
Pressure (4)	-.14*	.15*	-.24*
PSEC (5)	.77	-.03**	-.23*
**Building the full model**			
(1) + (2) + (3)	-	-	-.15
(1) + (2) + (3) + (4)	-	-	-.14
(1) + (2) + (3) + (4) + (5)	-	-	-.12

* p<0.05; ** p<0.01; *** p<0.001.

#### Mediation effects

**Figure 2:**
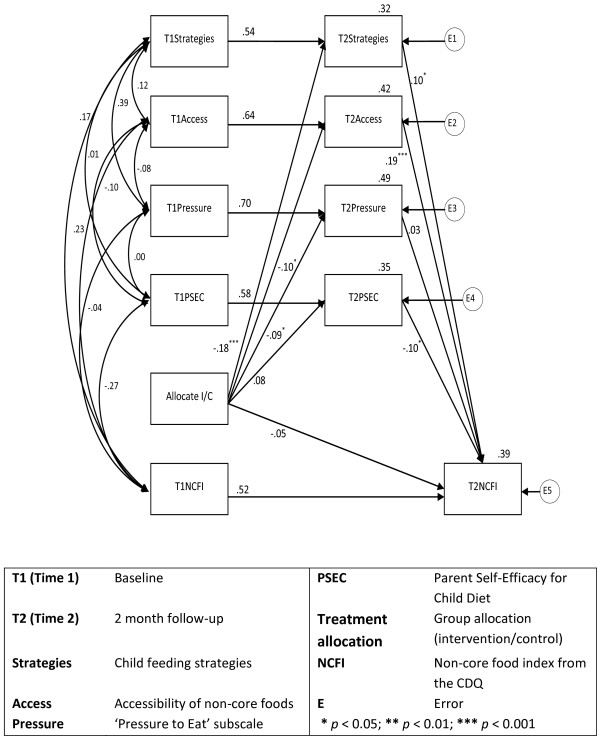
‘Full’ mediation model with standardised regression weights of pathways and the squared multiple correlations of variables.

**Figure 3:**
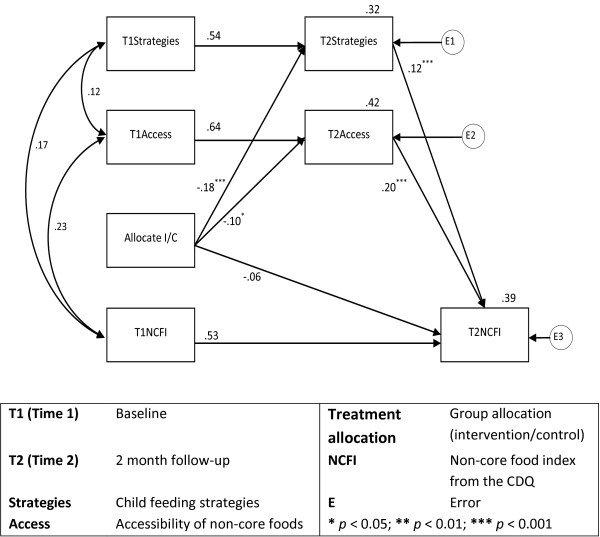
‘Final’ mediation model.

In the ‘full’ model (Figure 2) with four mediators, the strength of the relationship between treatment allocation and children’s non-core food consumption dropped from -.22 to -.05, and was no longer statistically significant, further reinforcing the role of the mediating variables in bringing about the change that occurred. In the full model the ‘treatment allocation’ variable (denoting participant allocation to intervention or control group) had a statistically significant negative relationship with ‘strategies’, ‘access’, and ‘pressure’, and these variables then had a positive effect on the outcome measure of children’s non-core food consumption (NCF). ‘Treatment allocation’ had a positive, though non-significant, relationship with ‘PSEC’, which had a significant negative effect on NCF. Whilst this model is the most explanatory, the most parsimonious ‘final’ model is presented in Figure 3, and includes the mediators ‘strategies’ and ‘access’. The figures show the standardised regression weights for each pathway in the model, providing an indication of the strength of effect between variables. The figures also show the squared multiple correlations (R^2^) presented above the each outcome variable, representing the percentage of the variance explained by the paths drawn to that variable. For example, in both Figures 2 and 3, children’s non-core food consumption (T2NCFI) has a squared multiple correlation of 0.39, indicating that 39% of the variance in the outcome is explained by the model.

## Discussion

On the basis of previous systematic reviews [1,16,20], this is one of very few randomised trials assessing the impact of a parent-based child nutrition intervention on the consumption of non-core foods among children aged 5 years and under. Furthermore, it represents the first study to examine the parent and home food environment characteristics which may mediate an intervention effect among children of this age. As such, the trial addresses important gaps in the child nutrition research and represents a novel contribution to scientific literature on this issue.

The baseline data indicate that children in both the control and intervention group were exceeding the recommended intake of non-core foods [6]. The trial found that at 2 months, the intervention was effective in reducing non-core food consumption among children whose parents were allocated to the intervention group relative to children whose parents were allocated to the control group. This effect remained significant under the conservative assumptions of an intention to treat analysis at 2 months. However, the intervention effect on child non-core food consumption appears to be short term, and was not maintained at 6 months. These findings are inconsistent with a number of past child nutrition interventions, which have sustained longer-term intervention effects on child dietary indices [8,20]. Further research investigating strategies capable of achieving long-term improvement in child non-core food consumption, such as extending the intensity and duration of telephone support, is therefore warranted.

Child access to non-core foods in the home, and child feeding strategies (e.g., restricting or rewarding with desert, finish dinner or seconds policies), were significant mediators, and as such represent the primary causal pathways (among those investigated) by which the intervention influenced the consumption of non-core foods at the 2 month follow-up. These findings confirm previous cross-sectional associations between child diet, access to foods, and child feeding strategies [45] and suggest that targeting accessibility and parent policies are likely to be an important part of childhood nutrition interventions and should also be considered for use by health practitioners. Parent self-efficacy was also found to be associated with child dietary intake, however, the intervention failed to significantly influence parent’s self-efficacy. Nonetheless greater attention to improving parent self-efficacy is likely to have a positive impact on child diet in future intervention trials.

A number of study limitations warrant consideration. The study relied on self-reported assessments of home food environment characteristics, and a short food frequency questionnaire to assess non-core food intake. The use of direct observation to assess the home food environment, and multiple 24 hour recall method to assess non-core food intake would have improved the internal validity of the study findings [46,47]. Further, assessments of the reliability and validity of some items used to assess non-core food intake, availability and accessibility of non-core foods in the home were modest. Additional methodological work to construct more psychometrically sound scales would represent an important contribution to future research in the field where more rigorous methods of data collection are not feasible. It is also possible that associations between the hypothesised mediating variables and non-core food score may have been confounded by factors not assessed in this study. Further, the trial did not assess parental or child weight status, a factor known to be associated with the trial outcome and some of the hypothesised mediating factors [48,49]. Inclusion of weight status should be considered as a possible explanatory variable in future studies. Finally the trial did not employ an attention control group. The extent to which the reported intervention effect was attributable to the content of the telephone contact rather than the telephone contact itself is not known. Notwithstanding these study limitations, the trial provides important information for researchers and practitioners regarding the impacts of a brief telephone-based intervention targeting children 3–5 years.

## Abbreviations

CDQ: Children’s dietary questionnaire; NCF: Non-core food subscale of the children’s dietary questionnaire; CATI: Computer assisted telephone interview system; PSEC: Parental self-efficacy for child diet scale; HHS: Healthy home survey; GEE: Generalised estimating equation; SEM: Structural equation modelling; GFI: Goodness of-fit; AGFI: Adjusted goodness of-fit; NFI: Normed fit index; RMSEA: Root mean square of approximation.

## Competing interest

The authors declare that they have no competing interests.

## Authors’ contributions

LW conceived the trial and secured grant funding. AF, RW, LW and JB developed the intervention. AF and SD developed study measures. LW, AF and RW managed recruitment, intervention implementation and data collection. AF and PM conducted the analysis. AF lead the drafting of the manuscript. All authors contributed to interpretation of analysis, provided critical comment on the manuscript draft and approved the final version.
